# Successful pembrolizumab treatment in a patient with ALK-positive lung adenocarcinoma: A case report and literature review

**DOI:** 10.1097/MD.0000000000043352

**Published:** 2025-07-11

**Authors:** Yugo Matsumura, Seiya Ichihara, Kaori Nii, Naoki Kadota, Yoshio Okano, Hisanori Machida, Nobuo Hatakeyama, Keishi Naruse, Tsutomu Shinohara, Eiji Takeuchi

**Affiliations:** a Department of Respiratory Medicine, National Hospital Organization Kochi Hospital, Kochi, Japan; b Department of Pathology, National Hospital Organization Kochi Hospital, Kochi, Japan; c Department of Community Medicine for Respirology, Graduate School of Biomedical Sciences, Tokushima University, Tokushima, Japan; d Department of Clinical Investigation, National Hospital Organization Kochi Hospital, Kochi, Japan.

**Keywords:** ALK-positive lung cancer, case report, immunotherapy, non-small cell lung cancer

## Abstract

**Rationale:**

Immune checkpoint inhibitors are generally considered to be ineffective in chemotherapy for anaplastic lymphoma kinase (ALK)-positive lung cancer. We encountered an ALK-positive lung cancer patient who responded well to pembrolizumab.

**Patient concerns:**

A 70-year-old man presented with a chest shadow.

**Diagnosis:**

A mass shadow in the left lower lung field was diagnosed as lung adenocarcinoma cT2N3M1b stage IVA. ALK staining by immunohistochemistry was strongly positive (3+), and fluorescence in situ hybridization detected ALK rearrangements. Immunostaining showed that 95% of tumor cells expressed programmed death ligand-1 (22C3 clones).

**Interventions:**

First-line therapy with alectinib was initiated, resulting in clinical improvement and a partial response (PR), which progressed after 14 months. The patient continued to receive various medications; however, the tumor gradually increased in size. Therefore, pembrolizumab was initiated as the 8th-line treatment and was effective.

**Outcomes:**

The tumor shrank after 2 cycles of pembrolizumab, and the patient achieved PR. Nine months later, the patient has maintained PR.

**Lessons:**

Few case reports have described the successful treatment of ALK-positive lung cancer with immune checkpoint inhibitors. We herein present a rare case of ALK-positive lung adenocarcinoma that responded to pembrolizumab monotherapy as the 8th-line treatment.

## 1. Introduction

Approximately 5% of non-small cell lung cancers (NSCLC) have anaplastic lymphoma kinase (ALK) rearrangements.^[1–4]^ Patients with ALK-positive NSCLC are more likely to be younger, nonsmokers, and diagnosed with more advanced disease than those with ALK-negative NSCLC.^[2,5,6]^ ALK inhibitors are first-line agents in chemotherapy for ALK-positive lung cancer.^[7–10]^ The treatment of lung cancer has been revolutionized by the advent of immune checkpoint inhibitors (ICIs). Nevertheless, the response rate of ALK-positive lung cancer to ICI monotherapy ranges between 0 and 8%; therefore, it is generally considered to be ineffective.^[11,12]^ Regarding ALK-positive lung cancer, median progression-free survival (PFS) for ALK-tyrosine kinase inhibitor-treatment-naive and posttreatment patients were 3.9 and 1.5 months, respectively, with limited efficacy when ICIs were administered.^[13]^ We herein report a rare case of ALK-positive lung adenocarcinoma that responded to pembrolizumab monotherapy as the 8th-line treatment.

## 2. Case report

**Figure 1.**
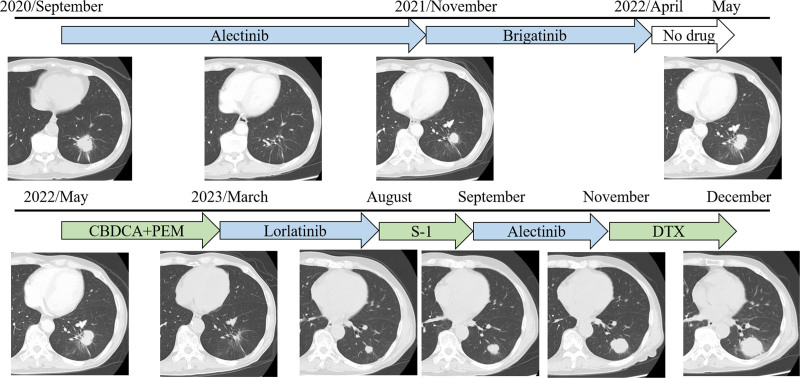
Clinical course of the patient. CBDCA + PEM = carboplatin and pemetrexed, TX = docetaxel, S-1 = tegafur gimeracil oteracil potassium.

**Figure 2.**
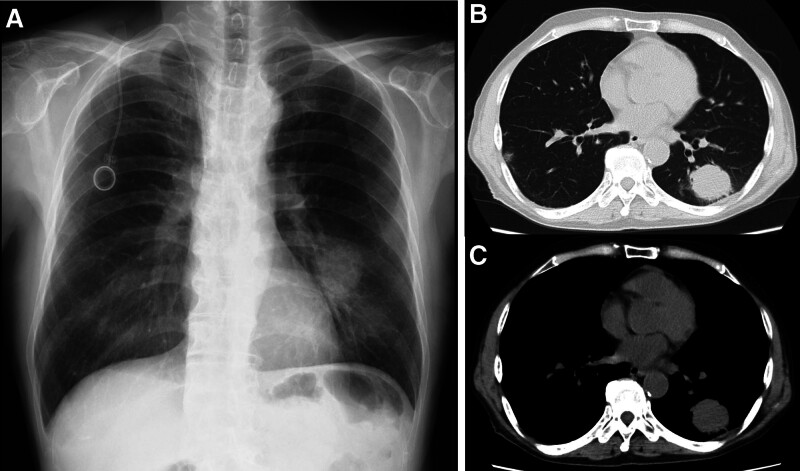
(A) Chest X-ray and (B and C) CT before the initiation of treatment with pembrolizumab: a 40-mm mass shadow in the left lower lobe. CT = computed tomography.

**Figure 3.**
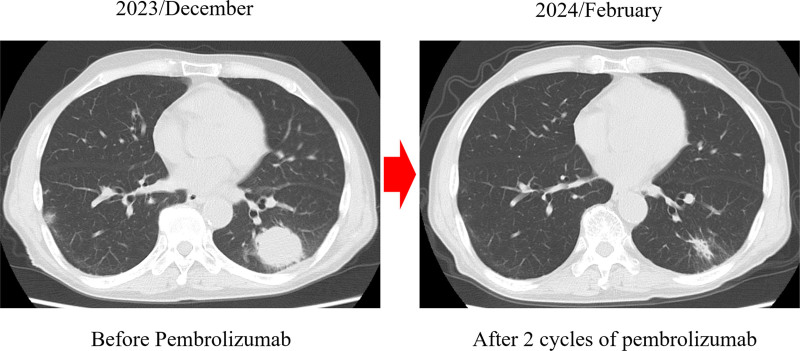
Computed tomography after pembrolizumab monotherapy. The tumor shrunk after 2 cycles, and the patient achieved a PR. PR = partial response.

A 70-year-old man was referred to our hospital with a chest shadow. His smoking habit was 1 pack of cigarettes a day for 30 years, he had a previous medical history of hypertension and chronic kidney disease, and there was no family history of lung cancer. The Eastern Cooperative Oncology Group performance status score was 1. The following blood examination results were obtained: white blood cell count 7130/μL, red blood cell count 286 × 10^4^/μL, platelet count 32.6 × 10^4^/μL, blood urea nitrogen 33.5 ng/dL, serum creatinine 1.13 mg/dL, and carcinoembryonic antigen 2.3 ng/mL. The neutrophil-to-lymphocyte ratio was 4.8. Chest X-ray and computed tomography (CT) revealed 35-mm mass shadows in the left lower lobe. Enlargement of the left hilar and mediastinal lymph nodes and ^18^F-fluorodeoxyglucose (FDG) uptake (maximum standardized uptake value = 7.1) were detected on FDG-positron emission tomography/CT. Adenocarcinoma was diagnosed from transbronchial lung biopsy specimens. Contrast-enhanced magnetic resonance imaging of the head showed brain metastasis in the right temporal lobe. Based on these findings, the patient was diagnosed with left lower lobe adenocarcinoma cT2aN3M1b stage IVA. ALK staining by immunohistochemistry was strongly positive (3+), and ALK rearrangements were confirmed by fluorescence in situ hybridization at a commercial laboratory (SRL, Tokyo, Japan). The expression of programmed death ligand-1 (PD-L1) in tumor cells was 95% by immunostaining (22C3 clones). First-line therapy with alectinib (600 mg/day) was initiated and resulted in clinical improvement and a partial response (PR). However, the tumor in the left lower lobe increased in size 14 months later. Although the patient continued to receive brigatinib, carboplatin and pemetrexed, lorlatinib, tegafur gimeracil oteracil potassium, alectinib, and docetaxel, the tumor gradually increased in size (Fig. 1). Chest X-ray and CT showed 40-mm mass shadows in the left lower lobes (Fig. 2). Therefore, pembrolizumab was initiated as the 8th-line treatment. The tumor shrunk after 2 cycles of pembrolizumab, and the patient achieved PR (Fig. 3). Nine months later, the patient has maintained PR.

## 3. Discussion

We encountered a rare case of ALK-positive lung adenocarcinoma that responded to pembrolizumab monotherapy as the 8th-line treatment. Based on a review of the literature, there have been 4 case reports of ALK-positive lung cancer that responded to ICI monotherapy.^[14–17]^

Approximately 5% of patients with NSCLC have ALK-positive disease.^[1–4]^ ALK-positive NSCLC patients are younger, in their 50s, nonsmokers, and more likely to be diagnosed with more advanced disease than those with ALK-negative NSCLC.^[2,4–6]^ ALK inhibitors are the first-line agents in chemotherapy for ALK-positive lung cancer.^[7–10]^ The treatment of lung cancer has been revolutionized by the advent of ICIs. Nevertheless, the response rate (RR) of ICI monotherapy for ALK-positive lung cancer ranges between 0 and 8%; therefore, it is generally considered to be ineffective.^[11,12]^ In ALK-positive lung cancer, median PFS is 3.9 months in ALK-TKI-naive patients and 1.5 months in posttreatment patients, with limited efficacy with ICIs.^[13]^

Table 1 shows a summary of the findings of the present and previous case reports. Among the 5 cases presented, mean age was 65 years and only 2 patients were female. Many cases had a smoking habit and high expression levels of PD-L1. Pembrolizumab was administered to 3 cases and nivolumab to 2. ICIs achieved a complete response in 1 patient and PR in 4. ALK-positive NSCLC patients who responded to ICIs were approximately 10 years older and more likely to be smokers than other ALK-positive NSCLC patients. In addition, the PFS for ALK-TKI was shorter.^[7–10]^ PD-L1 expression was high. However, PD-L1 is generally considered to be up-regulated in ALK-positive lung cancer and is not related to treatment efficacy.^[11,12,18,19]^ ALK-positive lung cancer patients who responded to ICIs were older, had a history of smoking, and had shorter PFS for initial ALK-TKI. ICIs may be effective in older patients who have a history of smoking and short PFS with initial ALK-TKI. Clinicians need to attempt treatment with ICIs for these patients at least once.

**Table 1:** ALK-positive lung cancer patients who responded to immune checkpoint inhibitors

No.	Author	Age (year)	Sex	Smoking history (pack-years)	PD-L1 TPS (%)	TKI	PFS (months)	ICIs	Response
1	Yamasaki^[14]^	76	Male	159	Unknown	Alectinib	5	Nivolumab	PR
2	Shimada^[15]^	51	Female	unknown	Unknown	Alectinib	5	Pembrolizumab	PR
3	Adachi^[16]^	78	Female	30	70	Alectinib	21	Pembrolizumab	PR
4	Baldacci^[17]^	48	Male	Never	100	Ceritinib	5	Nivolumab	CR
5	Our case	70	Male	30	95	Alectinib	14	Pembrolizumab	PR

ALK = anaplastic lymphoma kinase, CR = complete response, ICIs = immune checkpoint inhibitors, PD-L1 = programmed death ligand-1, PFS = progression-free survival, PR = partial response, TPS = tumor proportion score.

## 4. Conclusion

We herein presented a rare case of ALK-positive lung cancer that responded to ICI monotherapy as the 8th-line treatment.

## Author contributions

**Conceptualization:** Yugo Matsumura, Seiya Ichihara, Eiji Takeuchi.

**Data curation:** Kaori Nii, Naoki Kadota, Yoshio Okano, Hisanori Machida, Nobuo Hatakeyama, Keishi Naruse.

**Supervision:** Tsutomu Shinohara.

**Visualization:** Keishi Naruse.

**Writing – original draft:** Yugo Matsumura.

**Writing – review & editing:** Eiji Takeuchi.
